# Influence of Interfacial Traps on the Operating Temperature of Perovskite Solar Cells

**DOI:** 10.3390/ma12172727

**Published:** 2019-08-26

**Authors:** Hooman Mehdizadeh-Rad, Jai Singh

**Affiliations:** College of Engineering, IT and Environment, Charles Darwin University, Darwin NT 0909, Australia

**Keywords:** perovskite solar cells, operating temperature, interface passivation, degradation

## Abstract

In this paper, by developing a mathematical model, the temperature of PSCs under different operating conditions has been calculated. It is found that by reducing the density of tail states at the interfaces through some passivation mechanisms, the operating temperature can be decreased significantly at higher applied voltages. The results show that if the density of tail states at the interfaces is reduced by three orders of magnitude through some passivation mechanisms, then the active layer may not undergo any phase change up to an ambient temperature 300 K and it may not degrade up to 320 K. The calculated heat generation at the interfaces at different applied voltages with and without passivation shows reduced heat generation after reducing the density of tail states at the interfaces. It is expected that this study provides a deeper understanding of the influence of interface passivation on the operating temperature of PSCs.

## 1. Introduction

The power conversion efficiency (PCE) of perovskite solar cells (PSCs) has grown drastically during recent years, and a PCE of higher than 23% for single PSCs and about 28% for perovskite/silicon tandem solar cells have been reported recently [1,2,3,4,5,6]. One of the factors that can influence the PCE of solar cells is their temperature during the operation or the operating temperature T. It is well-known that if the operating temperature decreases then the diffusion length of charge carriers and PCE of PSCs increase for T > 200 K [7,8,9]. A high operating temperature may lead to the degradation in PSCs due to the decomposition of the active layer. Conings et al. [10,11,12] have investigated the thermal stability of PSCs and found that perovskite may decompose into PbI_2_ even at as low a temperature as 85 °C. Philippe et al. [11,12,13] have investigated the thermal stability of PSCs by maintaining them for 20 minutes at room temperature, 100 °C and 200 °C and observed that MAPbl3 starts to decompose into Pbl_2_ at 100 °C. They carried out this experiment under high vacuum conditions of 10^−8^ mbar. Also, it is found that the temperature becomes much too high at the points of creation of localized defects, which may lead to physical or chemical changes in any semiconductor device [14]. Another challenge with perovskites is that their crystal structure becomes unstable by increasing the temperature, leading to phase changes. For example, it is reported that the phase change from tetragonal to cubic can occur at around 327 K in PSCs [15,16,17]. However, methyl-ammonium (MA)-based perovskites show a higher phase stability in comparison to formamidinium (FA) [18,19]. Therefore, understanding and controlling the factors that may lead to an increase in the operating temperature of PSCs is crucial for increasing their efficiency and stability.

In this paper, only the non-radiative recombination in the tail-states is considered and the higher order Auger-type recombination is neglected. In Auger recombination, an excited pair of charge carriers recombines and the energy released is transferred non-radiatively to another charge carrier to excite it to higher energy states [20]. Thus, an Auger recombination is a secondary process and its rate of occurrence is usually much lower, unless the excitation density is very high. The eventual recombination of high-energy charge carriers in an Auger process may occur at any trapping centres, as considered here. Therefore, the non-radiative recombination as considered here where a charge carrier can be trapped at a trapping centre in the tail states is considered to be dominant [21,22,23,24]. It is known that in a PSC, the interfaces of the active layer-ETL (electron transport layer) and active layer-HTL (hole transport layer) are found to have more defects than within the active layer which act as trapping centres leading to non-radiative recombination [25,26]. It is shown that the hysteric J-V behaviour of PSCs can be attributed to several factors such as ferroelectricity, ion migration, unbalanced charge collection rates and trap recombination at the interfaces and grain boundaries [27,28,29,30,31,32]. However, as the non-radiative recombination generates heat, leading to an increase in the operating temperature of solar cells and may reduce PCE and stability of PSCs. Snaith et al. [33] have found that the cp-TiO_2_ ETL modified with C60-SAM could effectively passivate the formation of trap states at the interfaces, which reduces the non-radiative recombination and suppresses the J-V hysteresis in PSCs thus fabricated. Thote et al. [11] have achieved efficient and stable ZnO-based PSCs using a high-working pressure sputtering technique. This technique produces higher quality ZnO films with fewer surface defects compared with conventional sputtering or sol-gel ZnO solution processes. However, the influence of passivation of the interfaces on the operating temperature which may lead to phase transition and degradation in the active layer of PSCs has not yet been clearly understood.

In this paper, by assuming that the reduction in the density of tail states at the interfaces occurs due to passivation, a mathematical model is developed to calculate the operating temperature of PSCs. Our results show that by reducing the interfacial density of tail states, the operating temperature of PSCs can be decreased significantly at higher applied voltages. Thus, by passivating the interfaces in PSCs and hence reducing the operating temperature, the degradation effects and phase transitions may be prevented.

## 2. Methods

**Figure 1:**
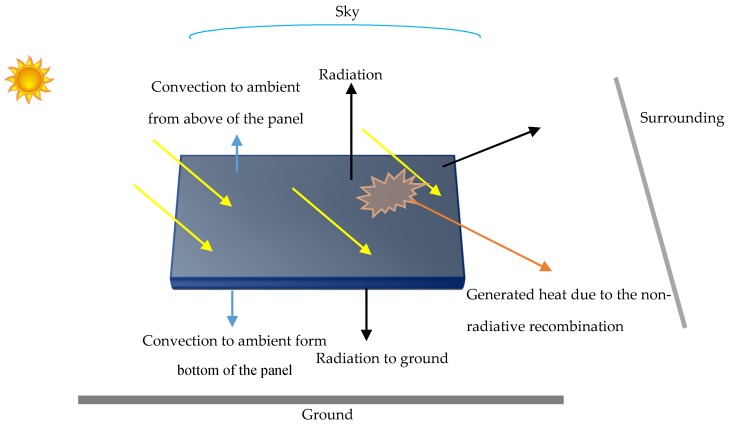
Schematic of heat transfer mechanisms in an illuminated solar cell: yellow arrows show incident solar power, orange arrow represents heat generation due to non-radiative recombination, blue arrows represent heat loss to the ambient air due to convection and black arrows represent heat loss due to radiation.

For an illuminated solar cell, the factors which may influence the operating temperature are solar radiation, heat generation due to the non-radiative recombination, wind velocity, ambient temperature and the heat transfer in solar cell’s material. An illuminated solar cell can transfer heat by radiation to sky, surroundings and ground and by convection to the ambient air. The thermal power generation (P) due to the non-radiative recombination in the active layer of an illuminated PSC can be considered as a heat source. Figure 1 presents different heat transfer mechanisms described above in an illuminated solar cell schematically.

**Figure 2:**
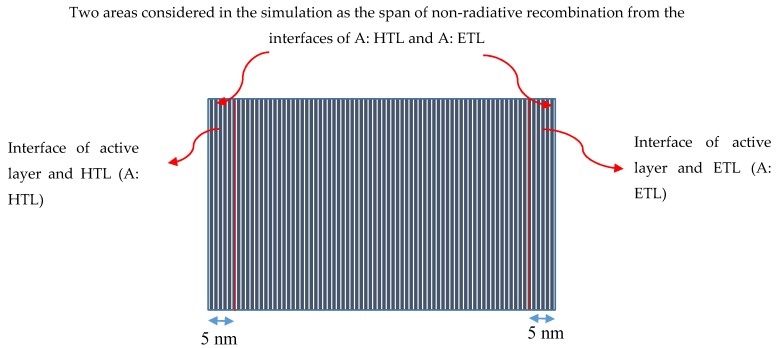
Division of the active layer of a PSC into meshes considered in the simulation. A distance of 5 nm from A: hole transport layer (HTL) and A: electron transport layer (ETL) interfaces into the active layer has been considered as the main areas of non-radiative recombination.

Although several simulations have been carried out by solving the drift diffusion equations, the effect of non-radiative recombination contributing to heat generation and hence, enhancing operating temperature in PSCs and organic solar cells has not yet been considered to the best of authors’ knowledge [27,34,35]. Therefore, in this paper, the temperature is considered as non-radiative recombination dependent and it is varied in the iteration of solving drift-diffusion equations. The simulation is started using an initial temperature which is changed after the first iteration and used as the initial temperature in the second iteration and so on until the convergence is achieved. For our simulation, the active layer of PSC is divided into meshes as shown in Figure 2. As the non-radiative recombination rate can be different at different points in the active layer, here, it is considered as position-dependent within the active layer starting from the HTL interface to the ETL interface, but it is assumed to be position independent in the lateral directions. Therefore, the heat generated power through the non-radiative recombination is considered to be position *x* dependent as *P*(*x*). However, as the Biot number is usually very small in thin films of perovskites, the heat gets distributed instantly in the active layer and the solar cell temperature can be assumed to be uniform within the whole active layer leading to the same temperature in all meshes considered in Figure 2. To show this, we have applied the lumped capacitance method for a PSC with the active layer CH_3_NH_3_PbI_3_ as discussed later in the Results and Discussion section.

For simulating the influence of the non-radiative recombination at the two interfaces of active layer and HTL (A: HTL) and active layer and ETL (A: ETL), it is assumed that the most non-radiative recombination may occur in an area within 5 nm in the perovskite active layer from each interface as shown in Figure 2.

**Figure 3:**
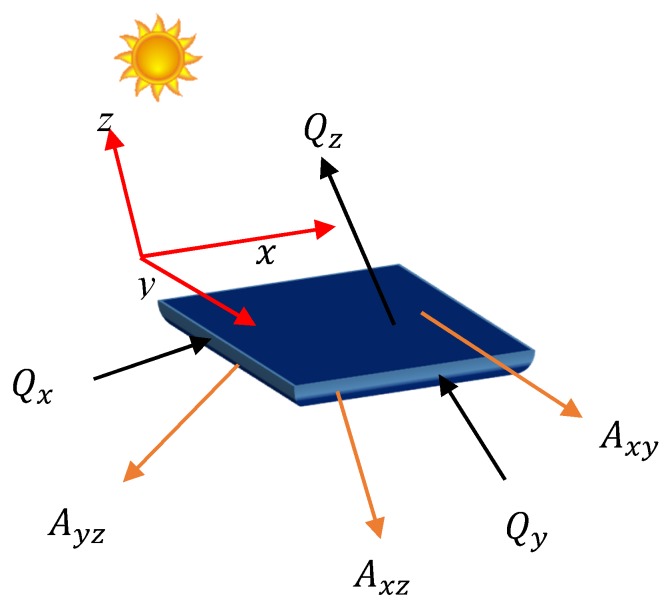
The conduction heat transfer directions in a solar cell.

It is also assumed that the heat transfer through conduction in the adjacent solar cells is negligible in a module. This assumption can be justified from the conduction heat transfer equations in the *x*-, *y*- and *z*-directions (*z-* towards the sun see Figure 3) given, respectively, by [36]:

(1) $$Q_{x}=kA_{yz}\frac{\partial T}{\partial x}$$

(2) $$Q_{y}=kA_{xz}\frac{\partial T}{\partial y}$$

(3) $$Q_{z}=kA_{xy}\frac{\partial T}{\partial z}$$

where $Q_{x}\;\left(Q_{y}\;\mathrm{and}\;Q_{z}\right)$ is the thermal energy transferred through conduction mechanism in the x (y and z) direction, and $A_{yz}\;(A_{xz}\;\mathrm{and}\;A_{xy})$ is the area of the lateral surface of the cell in the yz- (xz- and xy-) plane. $\frac{\partial T}{\partial x}\;\left(\frac{\partial T}{\partial y}\;\mathrm{and}\;\frac{\partial T}{\partial z}\right)$ is the gradient of temperature along the *x* (*y* and *z*) direction and k is the thermal conductivity of the solar cell material. According to Equations (1) and (2), in thin-film solar cells such as PSCs, as $A_{yz}$ and $A_{xz}$ are of the nanoscale and hence very small, leading to negligible conduction heat transfer ($Q_{x},\;Q_{y}\to 0$) towards the *x*- and *y*-directions. In addition, the conduction heat transfer along the z-axis is also negligible because the thickness of PSCs is of the nm scale, leading to the temperature gradient ($\frac{\partial T}{\partial z})$, which is negligibly small and hence, according to Equation (3), the conduction heat transfer along the *z*-direction becomes negligible ($Q_{z}\to 0)$. However, PSCs are encapsulated before being used and the effect of encapsulation should be considered in this analysis. As the thickness of encapsulation is only a few millimeters [37], the temperature gradient in the encapsulation layer can be neglected ($\frac{\partial T}{\partial z}\approx 0$, in the encapsulation layer). Therefore, it is justified to assume that the solar cell temperature and the temperature of the surface of the encapsulation are the same. This also leads one to assume that there is no air gap between the solar cell and encapsulation and then the only heat transfer from the surface of the solar cell to the encapsulation can occur through the conduction heat transfer but without the temperature gradient, this will be zero and thus no heat transfer may occur through the conduction.

In accordance with the above discussions, the operating temperature *T* of an illuminated PSC will depend on the radiation and convection heat transfers and non-radiative recombination of the photo excited charge carriers. Thus, we need to solve the following energy balance equation to determine T [36]:

(4) $$\begin{array}{c} Ir\alpha A_{xy}+P=h_{c,e-amb}A_{xy}\left(T-T_{amb}\right)+h_{r,e-sky}A_{xy}\left(T-T_{sky}\right)\\ +h_{r,e-ground}A_{xy}\left(T-T_{ground}\right)+h_{r,e-sur}A_{xy}\left(T-T_{sur}\right) \end{array}$$

where Ir is the incident solar radiation, α is absorption and P is the thermal power generated through the non-radiative recombination given by:

(5) $$P=R_{tail}E_{R}A_{xy}d$$

where $R_{tail}$ (m^−3^ s^−1^) is the rate of tail state recombination calculated by solving the Poisson and drift-diffusion equations [38,39,40], $E_{R}$ (eV) is the heat energy generated per recombination and d (nm) is the active layer thickness. $h_{c,e-amb}$ is convection heat transfer from encapsulation surface to ambient, $h_{r,e-sky}$, $h_{r,e-ground}$ and $h_{r,e-sur}$ in Equation (4) are the radiation heat transfer coefficients from encapsulation surface to sky, ground and surrounding, respectively, $T_{amb}$ is ambient temperature, $T_{sky}$ is sky temperature which can be determined by $T_{sky}=0.0552\;{T_{amb}}^{1.5}$ [41]. $T_{ground}$ and $T_{sur}$ are ground and surrounding temperatures which are considered equal to $T_{amb}$.

The radiation heat transfer coefficients from encapsulation surface to sky, ground and surrounding can be determined, respectively, by [36,42]:

(6) $$h_{r,e-sky}=\varepsilon_{c}\sigma_{sb}\left(T+T_{sky}\right)\left(T^{2}+{T_{sky}}^{2}\right)$$

(7) $$h_{r,e-ground}=\varepsilon_{c}\sigma_{sb}\left(T+T_{ground}\right)\left(T^{2}+{T_{ground}}^{2}\right)$$

(8) $$h_{r,e-sur}=\varepsilon_{c}\sigma_{sb}\left(T+T_{sur}\right)\left(T^{2}+{T_{sur}}^{2}\right)$$

where $\varepsilon_{c}$ is the emissivity coefficient of solar cell and $\sigma_{sb}=5.67\times 10^{-8}$ is the Stefan–Boltzmann constant. The convection heat transfer coefficient from encapsulation surface to the ambient air can be determined by [43]:

(9) $$h_{c,e-amb}=5.62+3.9v$$

where v is the wind velocity in the ambient.

**Figure 4:**
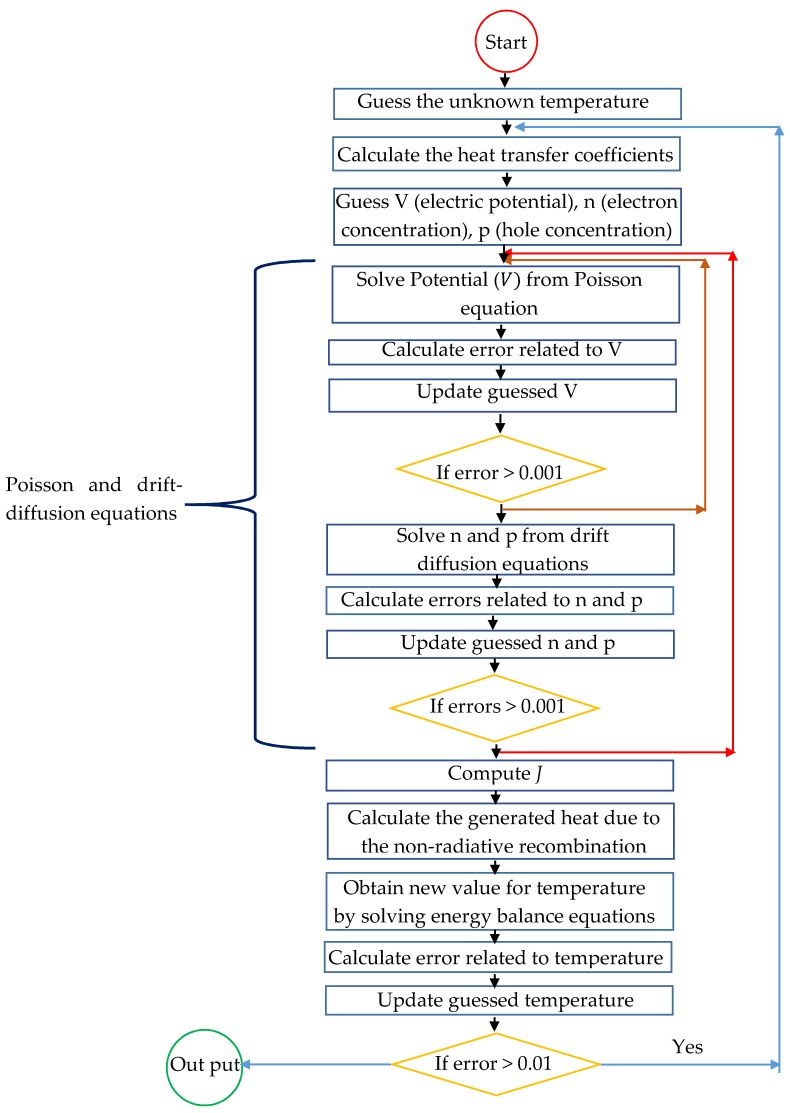
The data flow chart for solving the proposed simulation.

The thermal power generated through the non-radiative recombination in the illuminated PSC is found to be dominant [21,22,23,24] and can be considered as a heat source [44]. In the non-radiative recombination, it is assumed that one of the charge carriers (electron or hole) is trapped in the tail states and the other (electron or hole) is free in the conduction band (CB) or valence band (VB). Thus, sum of the thermal energy released due to the non-radiative recombination of free electrons in the CB with the trapped holes in the VB tail states, and free holes in VB with the trapped electrons in the CB tail states may be assumed to be equal to the band gap energy, i.e., $E_{R}\approx E_{g}$ in Equation (5). Using this in Equation (4), the temperature T can be determined by solving the following transcendental equation in *T*:

(10) $$\begin{array}{c} T=(Ir\alpha A_{xy}+R_{tail}E_{g}A_{xy}d+h_{c,e-amb}A_{xy}T_{amb}+h_{r,e-sky}A_{xy}T_{sky}\\ +h_{r,e-ground}A_{xy}T_{ground}+h_{r,e-sur}A_{xy}T_{sur})/(h_{c,e-amb}A_{xy}\\ +h_{r,e-sky}A_{xy}+h_{r,e-ground}A_{xy}+h_{r,e-sur}A_{xy} \end{array}$$

where $h_{c,e-amb}$, $h_{r,e-sky}$, $h_{r,e-ground}$ and $h_{r,e-sur}$ are used as a function of T given in Equations (6)–(8) and the rate of tail state recombination $R_{tail}$ is calculated by solving Poisson and drift-diffusion equations. We solve Equation (10) by iteration. First, we start with an initial temperature *T* to solve the drift-diffusion equations and calculate the heat transfer coefficients in Equations (6)–(8). Then, by substituting back these calculated $R_{tail}$, $h_{c,e-amb}$, $h_{r,e-sky}$, $h_{r,e-ground}$ and $h_{r,e-sur}$ in Equation (10), we determine the new solar cell temperature. The iteration is continued until the self-consistency is achieved. The above procedure of simulation of temperature is presented in the data flow chart as shown in Figure 4.

## 3. Results and Discussions

**Figure 5:**
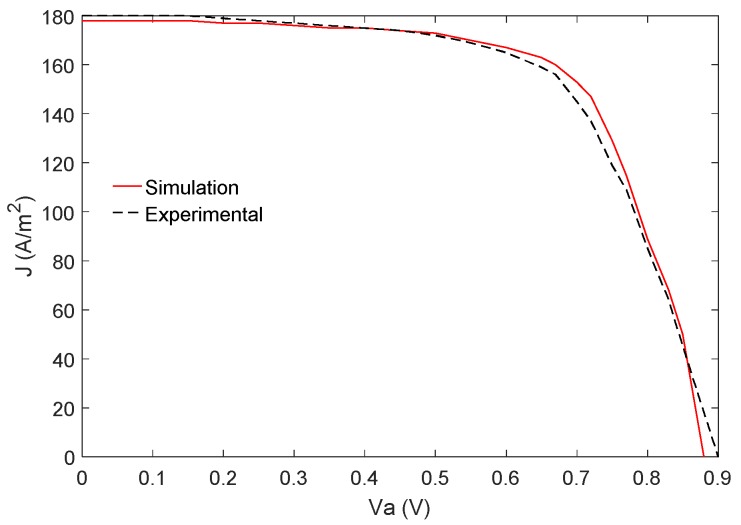
The J−V characteristics of a PSC of structure Glass/PEDOT: PSS/CH_3_NH_3_PbI_3_/PC60BM/Al obtained from our simulation (solid curve) and from experiment [45] (dotted curve) to check the validity of our simulation.

The simulation of the operating temperature of an illuminated PSC of the structure Glass/ITO/PEDOT: PSS/CH_3_NH_3_PbI_3_/PCBM/Al is presented here. However, first we would like to present the validation of our simulation by calculating the J-V characteristics of the above PSC considered in this paper and compare these with the experimental results measured by Kim et al. [45]. The input data required for the simulation of the J-V characteristics and operating temperature are listed in Table 1. The J-V characteristics obtained from the simulation are shown as a solid curve in Figure 5 along with the experimental results as the dotted curve. As it can be seen from Figure 5, our simulation results agree very well with the experimental ones.

**Figure 6:**
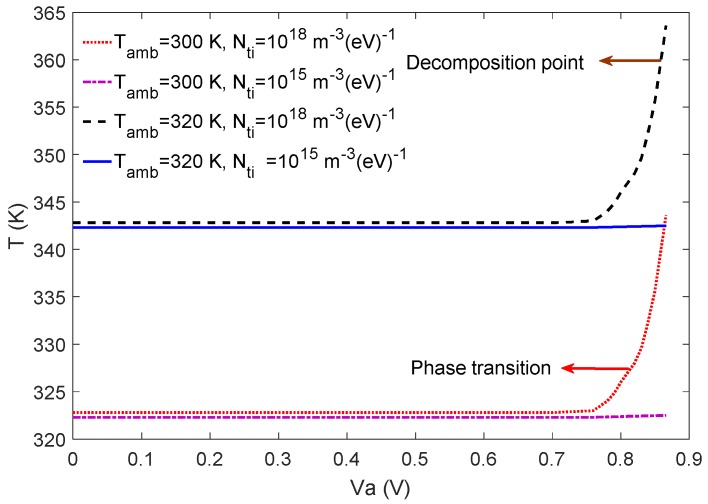
The operating temperature in the active layer plotted as a function of the applied voltage at two ambient temperatures of 300 K and 320 K.

In our simulation, following the observed density of tail states before and after the passivation at the interfaces by thermal admittance spectroscopy [24], it is assumed that the density of tail states at the interfaces *N_ti_* may reduce from 10^18^ to 10^15^ m^−3^ (*eV*)^−1^ passivating the interfaces. The operating temperature is calculated for *N_ti_* = 10^18^ and 10^15^ m^−3^ (*eV*)^−1^ at two different ambient temperatures of 300 K and 320 K and plotted as a function of the applied voltage $V_{a}$ as shown in Figure 6. According to Figure 6, for low applied voltages, $V_{a}\leq V_{max}$, where $V_{max}$ is the voltage at the maximum power point, it is found that the (i) operating temperature remains constant and (ii) influence of the density of tail states in the interface on the temperature of the solar cell is not very significant. It may be noted that in Figure 6, the maximum voltage is $V_{max}\approx 0.77$ V at the ambient temperature $T_{amb}=300\;K$ and $V_{max}\approx 0.75$ V at $T_{amb}=320\;K$. However, at $V_{a}\geq V_{max}$, the operating temperature increases by nearly 21 K at the $V_{oc}$ at both the ambient temperatures of 300 K and 320 K in the PSC without the passivation of the interfaces with the higher density of tail states *N_ti_* = 10^18^ m^−3^ (*eV*)^−1^. This is in contrast with the passivated PSC with the lower density of tail states *N_ti_* = 10^15^ m^−3^ (*eV*)^−1^ where the operating temperature remains nearly constant with the increase in the voltage. At the ambient temperature $T_{amb}$ = 300 K and applied voltage $V_{a}\approx$ 0.81 V, the temperature in the active layer of PSC without interface passivation increases to 327 K (red arrow), which is the temperature of phase transition in perovskite from tetragonal to cubic.

It may be noted that the decomposition of perovskite can be started at 358 K [10,11,12]. According to Figure 6, although the operating temperature of PSC without the interface passivation at the ambient temperature 300 K (red dotted curve) increases with applied voltage, it may never reach the decomposition temperature of 358 K because the maximum increase in temperature at the $V_{a}=V_{oc}$ is only about 343 K. However, at $T_{amb}=$ 320 K, the PSC without interface passivation may reach 358 K at $V_{a}\approx$ 0.85 V (black dashed curve) and may decompose, which will not occur in the passivated PSC. It should be mentioned that the $V_{oc}$ of solar cells decreases slightly by the increase in the ambient temperature.

It may be desirable to investigate the influence of thermalisation due to the non-radiative recombination on the open circuit voltage ($V_{oc})$, short circuit current ($J_{sc}$) and fill factor (FF). To address this issue, we have calculated $V_{oc}$, $J_{sc}$ and *FF* using the proposed iteration method by: (i) varying the operating temperature due to non-radiative recombination and (ii) keeping it constant equal to the ambient temperature during the iteration. The results obtained show that $V_{oc}$ decreases from 0.90 to 0.87 V and *FF* from 78% to 77% due to the increase in the operating temperature from the non-radiative recombination. However, $J_{sc}$ remains almost unchanged in both calculations. Thus, as expected, a slight reduction in $V_{oc}$ and *FF* are found due to the thermalisation effects caused by the non-radiative recombination.

**Figure 7:**
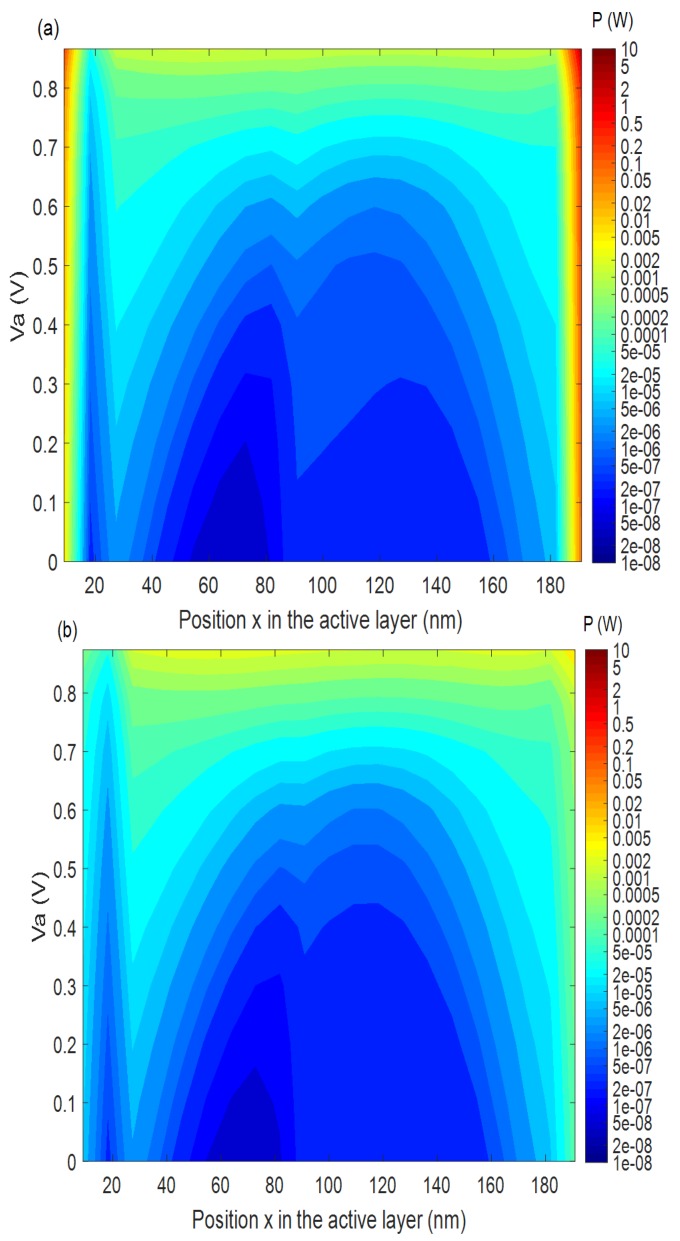
The contour plot of heat generation rate due to the non-radiative recombination as a function of position *x* in the active layer and applied voltage $V_{a}$ with (**a**) *N_ti_* = 10^18^ and (**b**) *N_ti_* =10^15^ m^−3^ (*eV*)^−1^.

In order to investigate the heat generation due to the non-radiative recombination at an applied voltage $V_{a}$ and at a position (*x*) in the active layer measured from the anode, we have shown the contour plots of the power generated by the non-radiative recombination *P* in Equation (5) as a function of the applied voltage $V_{a}$ and position *x* with *N_ti_* of 10^18^ and and 10^15^ m^−3^ (*eV*)^−1^ in Figure 7a and b, respectively. As it can be seen in Figure 7a, for *N_ti_* = 10^18^ m^−3^ (*eV*)^−1^ P increases when *x* approaches the interfaces at all the applied voltages, and becomes red in colour at the interfaces, which means that it becomes high at the interfaces. This is expected because more non-radiative recombinations occur at the interfaces and hence more heat generation at the interfaces. However, according to Figure 7b for *N_ti_* = 10^15^ m^−3^ (*eV*)^−1^, the power generation at the interfaces is much less (blue in colour), showing much less heat generation at the interfaces due to the passivation. It may be noted that the power *P* plotted in Figure 7a and b is nearly independent of the ambient temperature $T_{amb}$.

**Figure 8:**
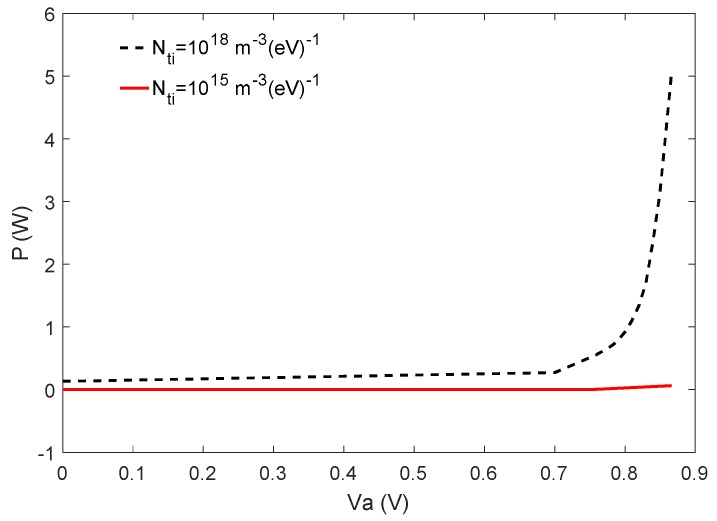
The total heat generation rate (P in W) due to the non-radiative recombination through the active layer as a function of the applied voltage $V_{a}$.

In order to calculate the total P through the active layer, we have integrated P over the active layer and the results are shown in Figure 8 at different applied voltages for *N_ti_* = 10^18^ m^−3^ (*eV*)^−1^ and 10^15^ m^−3^ (*eV*)^−1^. According to Figure 8, P is almost constant and close to 0 for *N_ti_* = 10^15^ m^−3^ (*eV*)^−1^ at the interfaces, while it grows to roughly 5 W by increasing the voltage of the cell with *N_ti_* = 10^18^ m^−3^ (*eV*)^−1^. Therefore, it may be concluded that at an ambient temperature higher than 300 K, PSCs may degrade faster without the passivation of the interfaces if subjected to a higher applied voltage.

As mentioned in the mathematical model section, by using a lumped capacitance method, we can assume the temperature of the solid is spatially uniform at any instant and the temperature gradient within the solid is negligible [36]. To validate this method, the Biot number, which is a dimensionless number for validation of the lumped capacitance method, should be less than 0.1 (Biot << 0.1). The Biot number can be determined by [36]:

(11) $$\mathrm{Biot}=\frac{L_{c}h_{c,c-amb}}{k}$$

where $L_{c}$ is characteristic length and can be determined by $L_{c}=Vol/A_{xy}$ and Vol is volume of solar cell. Also, we have calculated $h_{c,c-amb}$ by using Equation (9), and it is 5.62 to 44.62 ($W/m^{2}K)$ for wind velocities between 0 to 10 m/s. Heiderhoff et al. [46] have found that the thermal conductivity (*k*) of CH_3_NH_3_PbX_3_ single crystals with X = I, Br, and Cl is 0.34 ± 0.12, 0.44 ± 0.08, and 0.50 ± 0.05 W/(mK), respectively, at room temperature. By considering CH_3_NH_3_PbI_3_ with a thickness of 200 nm and with wind velocity = 10 m/s, the Biot $\approx 2.6\times 10^{-5}$, which is much less than 0.1. Therefore, the lumped capacitance method is effectively validated for a PSC. This implies that the temperature of the PSCs is spatially uniform at any instant, and the temperature gradient within the solar cell is negligible.

## 4. Conclusions

In this paper, the temperature in the active layer of a PSC before and after the interface passivation is simulated. It is found that by passivating the interfaces, which means by reducing the density of tail state recombination centres, the operating temperature of a PSC can be significantly lowered at higher applied voltages. Thus, the degradation of the active layer in PSCs can be reduced. It is shown that the operating temperature of a PSC can be lowered by 21 K by reducing the density of tail states at the interfaces by three orders of magnitude at the open circuit voltage condition. Such a reduction in the tail state densities at the interfaces may prevent phase change at the ambient temperature of 300 K, which may occur otherwise without the passivation. Also, it is shown that the decomposition of the active layer of a perovskite solar cell may be prevented at an ambient temperature of 320 K with the passivation.

## Figures and Tables

**Table 1:** Input parameters used for simulation in this paper [9].

Parameter	Value
$\varepsilon_{c}$	0.9
Ir $\left({\mathrm{wm}}^{-2}\right)$	1000
U (m/s)	0.1
$T_{amb}$ (K)	300
α	0.6
$E_{g}$ (eV)	1.5
d (nm)	200
$N_{c}$, $N_{v}$ ($m^{-3})$	$10^{26}$
$N_{ti}$ (density of tail state at interface) (($m^{-3}{\left(\mathrm{eV}\right)}^{-1}$)	$10^{15}$
$N_{ta}$ (density of tail state in the active layer) $(m^{-3}{\left(\mathrm{eV}\right)}^{-1}$)	$10^{14}$
$\mu_{n}\;\left(m^{2}V^{-1}s^{-1}\right)$	$0.5\times 10^{-4}$
$\mu_{p}\;\left(m^{2}V^{-1}s^{-1}\right)$	$0.5\times 10^{-4}$
$\beta_{n}^{0}$ (${\mathrm{cm}}^{3}s^{-1})$	2.5 $\times 10^{-10}$
$\beta_{p}^{0}$ (${\mathrm{cm}}^{3}s^{-1})$	5 $\times 10^{-10}$
$E_{Uc}=E_{Uv}$ (meV)	45
